# More than royal food - *Major royal jelly protein* genes in sexuals and workers of the honeybee *Apis mellifera*

**DOI:** 10.1186/1742-9994-10-72

**Published:** 2013-11-27

**Authors:** Anja Buttstedt, Robin FA Moritz, Silvio Erler

**Affiliations:** 1 Institut für Biologie, Zoologie - Molekulare Ökologie, Martin-Luther-Universität Halle-Wittenberg, Hoher Weg 4, 06099 Halle, Saale, Germany; 2 Department of Zoology and Entomology, University of Pretoria, Pretoria 0002 South Africa; 3 Facultatea de Zootehnie şi Biotehnologii, Universitatea de Ştiinţe Agricole şi Medicină Veterinară, Calea Mănăştur 3-5, 400372 Cluj-Napoca, Romania

**Keywords:** Social insect, Queen determination, Caste determination, Caste system, MRJP, Apalbumin, Royalactin

## Abstract

**Background:**

In the honeybee *Apis mellifera*, female larvae destined to become a queen are fed with royal jelly, a secretion of the hypopharyngeal glands of young nurse bees that rear the brood. The protein moiety of royal jelly comprises mostly major royal jelly proteins (MRJPs) of which the coding genes (*mrjp1-9*) have been identified on chromosome 11 in the honeybee’s genome.

**Results:**

We determined the expression of *mrjp1-9* among the honeybee worker caste (nurses, foragers) and the sexuals (queens (unmated, mated) and drones) in various body parts (head, thorax, abdomen). Specific *mrjp* expression was not only found in brood rearing nurse bees, but also in foragers and the sexuals.

**Conclusions:**

The expression of *mrjp1* to *7* is characteristic for the heads of worker bees, with an elevated expression of *mrjp1-4* and *7* in nurse bees compared to foragers. *Mrjp5* and *6* were higher in foragers compared to nurses suggesting functions in addition to those of brood food proteins. Furthermore, the expression of *mrjp9* was high in the heads, thoraces and abdomen of almost all female bees, suggesting a function irrespective of body section. This completely different expression profile suggests *mrjp9* to code for the most ancestral major royal jelly protein of the honeybee.

## Introduction

The cornerstone of eusociality in insects is the caste differentiation [1]. Female individuals are polymorphic with functional and/or morphological different castes resulting in division of labour particular for reproduction. The basics of caste determination are best elucidated for the western honeybee *Apis mellifera.* Its female caste system with up to 80,000 sterile worker bees and a single queen in the colony is a textbook classic [2]. Female larvae either develop into a queen or a worker bee depending on the quality and the quantity of the diet provided by the nurse bees. Royal larvae exclusively receive royal jelly (RJ), a secretion of the hypopharyngeal glands of nurse bees that rear the brood [3], whereas pollen and honey is added to the diet of worker destined larvae.

Besides water (60-70%) RJ consists of 10-16% sugar, 12-15% crude protein, 3-6% lipids and traces of salts, free amino acids and vitamins [4,5]. The protein moiety comprises primarily major royal jelly proteins (MRJPs) of which nine different encoding genes have been identified (*mrjp1*-*9*) ([6-9]; for a review see [10]). Genes encoding MRJPs are not only found within the genus *Apis*, and are common in other Hymenopteran species including the solitary, parasitoid jewel wasp *Nasonia vitripennis*, the alfalfa leafcutter bee *Megachile rotundata*, as well as several bumble bees and ants ([10] and references therein). *Mrjps* are also found in the primitively eusocial paper wasp *Polistes canadensis *[11], representing the first *mrjp-*exhibiting species in the family Vespidae. The ancestral state of the genes seems to be the single copy condition [10,12]. Nevertheless, few cases of copy number radiations have occurred in Hymenopteran species including the parasitic wasp *N. vitripennis,* the fungus-growing ant *Acromyrmex echinator*, the argentine ant *Linepithema humile* and the leaf cutter ant *Atta cephalotes *[10]. The evolution of *mrjps* is well understood and they originated by duplication from *yellow* genes, a group of genes common throughout arthropods [7,13]. The *yellow* gene was originally identified as a *Drosophila melanogaster* mutation interfering with the melanic cuticle pigmentation [14]. Subsequently *yellow* genes were shown to be associated with sex-specific reproductive maturation and behaviour [13,15].

All MRJPs, except for MRJP8, have been detected in RJ or the hypopharyngeal glands of *A. mellifera *[9,16-25]. The genes *mrjp1* to *5* have been suggested to be primarily expressed in the hypopharyngeal glands of nurses but not in those of foragers [7,26-31]. This matched well with the notion that MRJPs are primarily a nutrient for the developing larvae [9,32,33]. In addition, MRJPs have been repeatedly studied because of their potential role as a queen determinator in RJ. However, many of these studies are inconclusive and not in line with each other. MRJP1 [34] and MRJP3 [35] have been claimed to induce larvae growth and function as queen determinators. However, also changing glucose and fructose concentrations [36,37] or the unsaturated fatty acid 10-hydroxy-2-decenoic acid [38] interfered with caste determination. In general it seems clear that the ancestral MRJP did not evolve to a caste determination protein in general in social insects. For example the genome of the solitary wasp *Nasonia vitripennis* which has no workers comprises a suite of 10 *mrjp* genes. Furthermore, given that *mrjp* genes are also present in e.g., the red harvester ant *Pogonomyrmex barbatus* which shows genetic caste determination based on an individual's genotype [39], it seems to be unlikely that the ancient MRJP functions as ‘queen determinator’. So if any MRJP is involved in environmental queen determination, this must be a very specific function that exclusively evolved in *Apis*.

The expression of *mrjp1-8* has been repeatedly shown also in the brain of nurse bees [29,40-44], and *mrjp1* and *3* were shown to be expressed in drones (head, body, larvae) and queens (ovary, larvae) [7]. MRJP1 to 3 were also found in the haemolymph of larvae [45] with a significant higher amount than in pupae haemolymph [46] and they were shown to be down-regulated or depleted after infection with a severe pathogen of the honeybee, *Paenibacillus larvae*[47]. Hence, MRJPs seem to have important functions for honeybee physiology, development and colonial organization in general [10,44] and not just as a food compound in RJ.

In this study we provide a comprehensive analysis on *major royal jelly protein* gene expression in all honeybee castes and both sexes (workers (nurses, foragers), queens (unmated, mated) and drones) in various body parts (head, thorax, abdomen) to assess to what extent expression of these genes is affected by caste and sex.

## Results

### Confirmation of primer specificity and validation of reference genes

The specificity of qPCR products was documented with the high-resolution automatic capillary electrophoresis system QIAxcel® (Qiagen, Hilden, Germany). No primer-dimers or unspecific products were generated and all *mrjp* transcripts resulted in single products with the predicted size (Additional file 1: Figure S1). In addition, melting curve analyses resulted in the corresponding specific melting temperatures (Additional file 1: Table S1).

Two genes, *RpS5a* and *arp1*, were analyzed in regard to their suitability to serve as reference genes. According to Pfaffl et al. [48], any studied gene with a C_t_ value standard deviation higher than 1 can be considered as inconsistent. Whereas *RpS5a* showed a standard deviation of 0.86, *arp1* had with 1.55 a much higher value. Thus, *arp1* was coregulated within the different castes, sexes and body sections and excluded as reference gene from further analyses.

### Tissue specific gene expression

**Figure 1:**
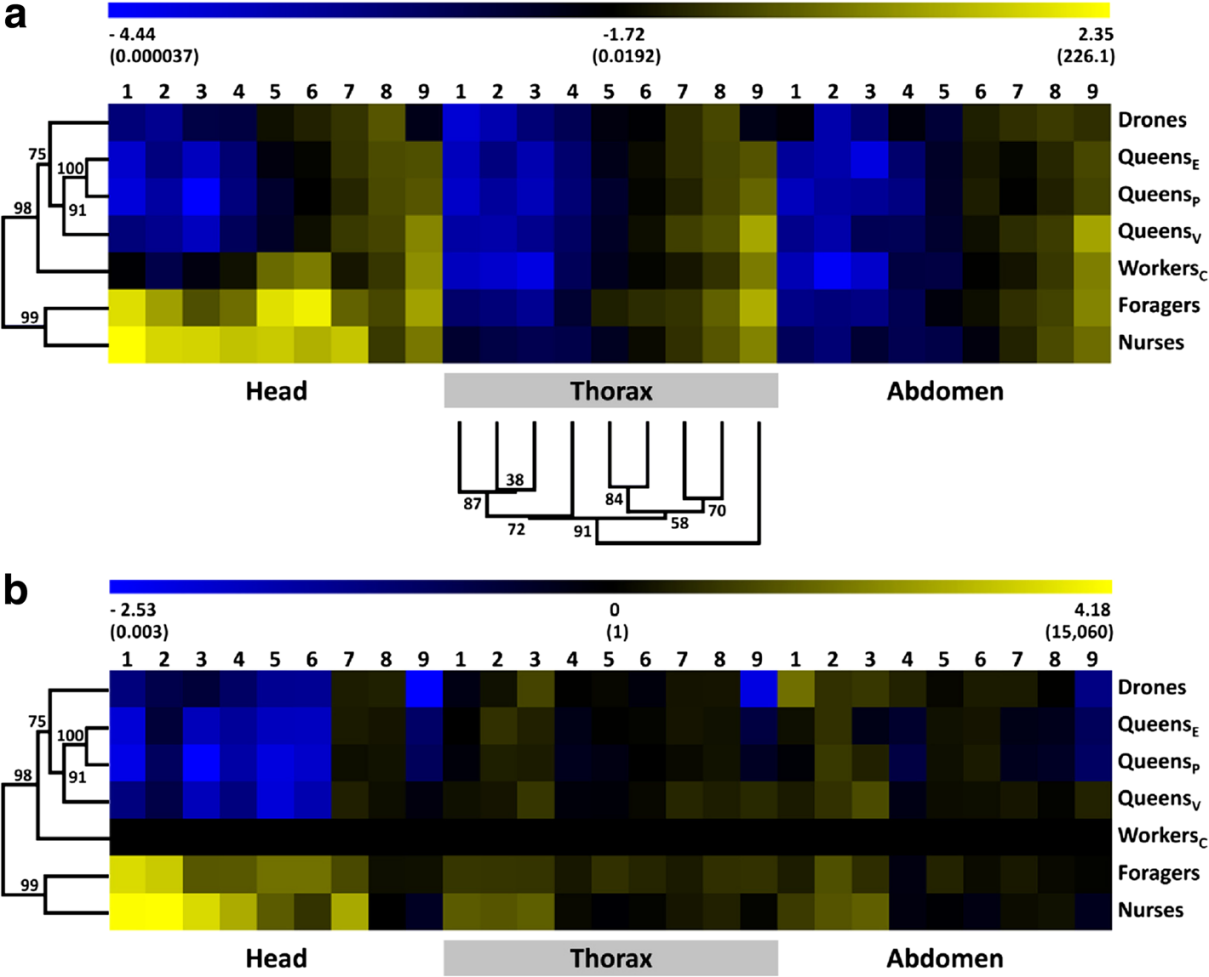
**Two-way hierarchical clustering analysis heat map and dendrogram of *mrjp *gene expression data over all honeybee castes, sexes and groups.** Branch lengths in dendrograms produced from cluster analysis correspond to the relative degree of similarity between branches. Differential gene expression is represented for all genes as a color gradient across all samples from deep blue (lowest) to light yellow (highest). The expression values were log transformed and visualized using MultiExperiment Viewer (MeV) version 4.9. **(a)** Relative gene expression across all honeybee groups and body sections. The dendrogram for the clustering of the *mrjps* was generated using data of all three body sections and is exemplarily shown for the thorax. The complete heat map that served as base for the clustering of the genes is shown in Additional file 1: Figure S2. Relative gene expression values are shown in brackets. **(b)** Gene expression normalized to caged workers. The normalized gene expression values are shown in brackets.

**Figure 2:**
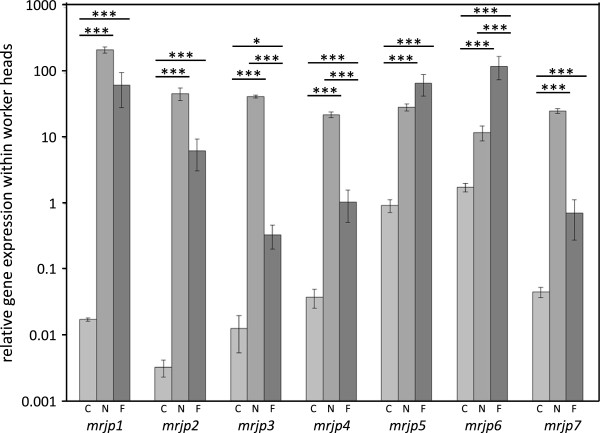
**Analyses of *mrjp1 *to *7 *mRNA levels in heads of worker honeybees.** The y-axis (log-scaled) indicates the relative levels of mRNA expression. Significant differences are marked by asterisks (* P < 0.05, *** P < 0.001). C, caged workers, light grey; N, hive nurses, grey; F, foragers, dark grey. *Mrjp8* and *9* are not shown as these genes are not specifically up-regulated within the head.

**Figure 3:**
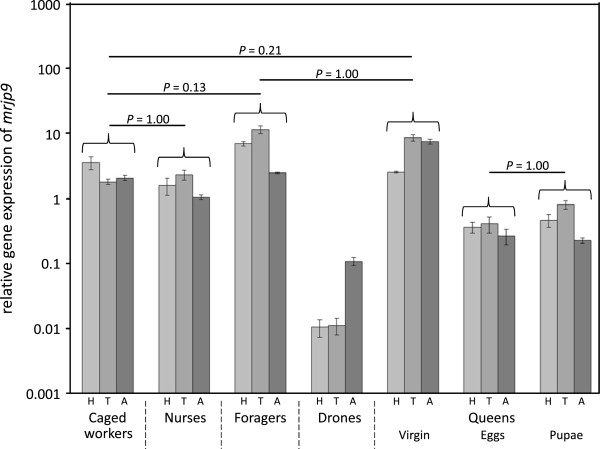
**Analyses of *mrjp9 *mRNA levels throughout honeybee castes, sexes and body sections.** The y-axis (log-scaled) indicates the relative levels of mRNA expression. Non-significant differences are marked by corresponding *P* values. All other between castes and sexes comparisons were significantly different (*P* < 0.01). H, head, light grey; T, thorax, grey; A, abdomen, dark grey.

*Mrjp* transcript abundance was highly variable among the various groups and tissues (Figure 1, Table 1). Within the drones and queens *mrjp* transcript abundance was generally low in all tissues, particulary for *mrjp1* to *5* (Figure 1a, Table 1). *Mrjps1* to *7* showed significantly increased transcript abundances in heads of nurses (N) and foragers (F), compared to caged workers (C), drones (D) and queens (Q) (Figure 2 and Additional file 1: Table S2). This drastic different gene expression leads to the division of the dendrogram into two different clusters, one comprising foragers and nurses and the other one the sexuals (Figure 1). *Mrjp8* was very evenly expressed in almost all analyzed groups and body sections (mean ± SE: 0.25 ± 0.03) suggesting a function independent of caste, sex and tissue. *Mrjp9* showed a completely different expression pattern from all of the other *mrjps*, with a significant increase in the expression in all worker bees and virgin queens compared to drones and mated queens (Figure 3), leading to a separate cluster in the dendrogram (Figure 1a). Except for drones, *mrjp9* was strongly expressed in the thorax and abdomen of all groups and always among the genes with the highest transcript abundances (Figure 1a and Additional file 1: Table S3).

**Table 1:** **Overview of relative *major royal jelly protein *** **(*mrjp*) gene expression levels (normalized to *RpS5a*) between honeybee castes, sexes and body sections**

		***mrjp1***	***mrjp2***	***mrjp3***	***mrjp4***	***mrjp5***	***mrjp6***	***mrjp7***	***mrjp8***	***mrjp9***
		**Mean (SE)**	**Mean (SE)**	**Mean (SE)**	**Mean (SE)**	**Mean (SE)**	**Mean (SE)**	**Mean (SE)**	**Mean (SE)**	**Mean (SE)**
**C**	H	0.01724	0.00322	0.01248	0.03744	0.95111	1.79112	0.04499	0.13981	3.49597
		(0.00099)	(0.00094)	(0.00713)	(0.01210)	(0.21579)	(0.26061)	(0.00815)	(0.00366)	(0.78429)
	T	0.00019	0.00014	0.00008	0.00220	0.00950	0.02362	0.04599	0.11695	1.75857
		(0.00002)	(0.00003)	(0.00004)	(0.00054)	(0.00146)	(0.00372)	(0.00985)	(0.00766)	(0.16579)
	A	0.00024	0.00005	0.00014	0.00384	0.00368	0.02055	0.04094	0.14514	2.02138
		(0.00005)	(0.00001)	(0.00002)	(0.00037)	(0.00009)	(0.00188)	(0.00778)	(0.00504)	(0.19282)
**N**	H	226.081	48.5296	43.4218	23.0384	29.9070	12.3185	26.2650	0.14957	1.53987
		(23.9031)	(10.6178)	(2.14839)	(2.37269)	(3.69670)	(3.11597)	(2.29565)	(0.06601)	(0.45245)
	T	0.00600	0.00358	0.00274	0.00321	0.00851	0.02886	0.10930	0.42138	2.25887
		(0.00203)	(0.00143)	(0.00123)	(0.00072)	(0.00173)	(0.00789)	(0.00601)	(0.12606)	(0.37526)
	A	0.00219	0.00115	0.00546	0.00270	0.00331	0.01321	0.06877	0.29266	1.02507
		(0.00026)	(0.00055)	(0.00143)	(0.00022)	(0.00068)	(0.00286)	(0.00167)	(0.07844)	(0.09004)
**F**	H	65.6187	6.47471	0.33646	1.06951	69.9339	127.812	0.71992	0.26347	6.83439
		(36.1326)	(3.25996)	(0.13335)	(0.55163)	(25.4869)	(49.3559)	(0.44058)	(0.02392)	(0.51357)
	T	0.00147	0.00103	0.00057	0.00564	0.05878	0.09711	0.12546	0.69147	11.1988
		(0.00088)	(0.00029)	(0.00009)	(0.00045)	(0.00655)	(0.00987)	(0.01608)	(0.07890)	(1.54652)
	A	0.00071	0.00096	0.00079	0.00259	0.01393	0.03328	0.10671	0.21950	2.41244
		(0.00025)	(0.00018)	(0.00010)	(0.00037)	(0.00117)	(0.00286)	(0.00992)	(0.01655)	(0.06201)
**D**	H	0.00104	0.00059	0.00343	0.00392	0.03592	0.06626	0.13269	0.45377	0.01031
		(0.00045)	(0.00015)	(0.00306)	(0.00011)	(0.00276)	(0.00645)	(0.03480)	(0.00669)	(0.00314)
	T	0.00012	0.00027	0.00106	0.00245	0.01321	0.01724	0.10092	0.26064	0.01095
		(0.00003)	(0.00005)	(0.00065)	(0.00082)	(0.00515)	(0.00578)	(0.04444)	(0.06591)	(0.00317)
	A	0.01627	0.00030	0.00117	0.01392	0.00501	0.05750	0.10938	0.16084	0.10576
		(0.00936)	(0.00008)	(0.00036)	(0.00552)	(0.00100)	(0.01227)	(0.03726)	(0.01107)	(0.01475)
**Q**_**V**_	H	0.00101	0.00064	0.00019	0.00214	0.00746	0.03413	0.15427	0.24149	2.48325
		(0.00084)	(0.00032)	(0.00007)	(0.00013)	(0.00264)	(0.00237)	(0.01255)	(0.03363)	(0.06404)
	T	0.00037	0.00032	0.00061	0.00175	0.00764	0.03337	0.19903	0.35160	8.42191
		(0.00004)	(0.00004)	(0.00018)	(0.00013)	(0.00322)	(0.00593)	(0.01759)	(0.05914)	(0.93819)
	A	0.00066	0.00031	0.00244	0.00237	0.00612	0.03503	0.09825	0.17340	7.38905
		(0.00008)	(0.00004)	(0.00030)	(0.00014)	(0.00115)	(0.00276)	(0.00210)	(0.01517)	(0.64135)
**Q**_**E**_	H	0.00014	0.00088	0.00019	0.00117	0.01314	0.02427	0.12057	0.31266	0.35568
		(0.00008)	(0.00032)	(0.00004)	(0.00017)	(0.00324)	(0.00203)	(0.02491)	(0.02422)	(0.06774)
	T	0.00020	0.00087	0.00028	0.00130	0.01030	0.02921	0.10260	0.23567	0.39935
		(0.00004)	(0.00014)	(0.00014)	(0.00043)	(0.00373)	(0.00051)	(0.00657)	(0.00233)	(0.11280)
	A	0.00046	0.00030	0.00008	0.00135	0.00730	0.04612	0.02465	0.07809	0.26115
		(0.00031)	(0.00006)	(0.00008)	(0.00039)	(0.00506)	(0.02421)	(0.00466)	(0.03510)	(0.07251)
**Q**_**P**_	H	0.00010	0.00039	0.00004	0.00091	0.00635	0.01924	0.07684	0.28650	0.45551
		(0.00003)	(0.00010)	(0.00004)	(0.00002)	(0.00110)	(0.00253)	(0.00810)	(0.02399)	(0.09806)
	T	0.00015	0.00044	0.00022	0.00114	0.00625	0.02365	0.06830	0.24382	0.78350
		(0.00003)	(0.00007)	(0.00008)	(0.00031)	(0.00209)	(0.00592)	(0.00983)	(0.00951)	(0.11533)
	A	0.00020	0.00041	0.00050	0.00084	0.00698	0.05543	0.02091	0.06242	0.22055
		(0.00003)	(0.00006)	(0.00016)	(0.00018)	(0.00138)	(0.02605)	(0.00190)	(0.00923)	(0.01979)

C, Caged workers; N, Hive nurses; F, Foragers; D, Drones; Q_V_, Virgin queens; Q_E_, Queens with eggs; Q_P_, Queens with pupae; H, Head; T, Thorax; A, Abdomen.

### Expression in workers

To better visualize the different regulation of *mrjps* especially among the worker caste, Figure 1b shows the expression data normalized to caged workers, which had a protein poor diet and could not activate food glands. Those workers showed only a basic expression of *mrjp1* to *7* in the head compared to nurse bees in the hive (Figure 1b and Additional file 1: Table S2). The observed up-regulation in nurses was fair for *mrjp 5* and *6* (30- and 6-fold, respectively; *P* < 0.001, one-way ANOVA, Bonferroni post-hoc test), higher for *mrjp4* and *7* (both 600-fold, *P* < 0.001) and tremendous for *mrjp3* (3,000-fold, *P* < 0.001), *mrjp2* (15,000-fold, *P* < 0.001) and *mrjp1* (13,000-fold, *P* < 0.001) (Figure 2).

Surprisingly, this general increase in the expression of the seven *mrjp* genes was not only found in nurses but also in foragers (Figure 2 and Additional file 1: Table S2). *Mrjps 1, 2, 5* and *7* were significantly up-regulated in foragers compared to caged workers (*P* < 0.001) but showed no significant difference to the up-regulation in nurses. The expression level of *mrjp6* was even 10-fold higher in foragers compared to nurses (*P* < 0.001). The increase in *mrjp3* expression in foragers (25-fold, *P* < 0.001) was significantly lower compared to the 3,000-fold increase in nurses (*P* < 0.001). This reduced increase was also found for *mrjp4*, which was only 28-fold higher in foragers, but 600-fold higher in nurses compared to caged workers (*P* < 0.001). In all three types of worker bees, *mrjp1* to *7* were only slightly expressed in thorax and abdomen, with an apparent minimal expression of *mrjp1* to *4* (relative gene expression below 0.01, Figure 1a, Table 1). The expression of *mrjp8*, similar in head and abdomen among the three worker types, differed only in the thorax with a 4- to 5-fold increased expression level in foragers and nurses compared to caged workers (*P* < 0.01) (Figure 1b). *Mrjp9* showed no difference between caged workers and nurses but the expression levels in forager head and thorax were 2- to 6-fold higher (P < 0.001).

### Expression in sexuals - queens and drones

There were no significant differences in transcript abundance for any of the *mrjp* genes between the two types of mated queens (Figure 1 and Additional file 1: Table S2). Virgin queens differed from mated queens primarily in *mrjp9*, with a 5- to 30-fold increased expression in all body parts (*P* < 0.001, one-way ANOVA, Bonferroni post-hoc test, pooled mated queens) (Figure 3). *Mrjp1* to *5* were only marginally expressed in all body sections of the three queen types with a relative expression value ≤ 0.01 (Figure 1a, Table 1).

The drones generally had the lowest *mrjp* expression levels. The highest expressed *mrjp* is with a relative expression of 0.29 ± 0.05 (mean ± SE) *mrjp8*, the one that is barely regulated and consistently expressed over all groups (Figure 1, Table 1). All of the other *mrjps* are even lower expressed with a relative expression ≤ 0.1. The analysis of *mrjp* expression profiles shows that drones cluster together with the three types of queens in one cluster far from nurses and foragers (Figure 1).

## Discussion

*Mrjps* are expressed in all body sections of both sexes and all castes of the honeybee *Apis mellifera* suggesting that the functions of these proteins are wide-ranging and not only constrained to the food glands. Our results show that the focus of the expression of *mrjp1* to *7* lies clearly in the worker heads (Figure 1). This is in accordance with previous studies confirming the association of some MRJPs with the activated food glands (for a recent review see [10]). Since the food glands are absent in drones and queens and not developed in the caged workers it is not surprising to see *mrjp 1* to *7* only minimal expressed (Figure 1). In the thorax and abdomen, the expression of the genes is similar low but not absent, showing that *mrjps* are involved in more than just producing nutritional protein.

In the recently compiled honeybee protein atlas of Chan et al. [49] MRJPs were shown to occur in various organs of the abdomen but not in the thoracic muscle and the thoracic salivary gland (see also [25]). Nevertheless, various MRJPs, except for MRJP4, 6 and 8, were identified in the honeybee’s nerve chord [49] that is crossing the thorax and abdomen [3]. Therefore, the basic expression of some *mrjps* in the thorax might be caused by expression in the nerve chord ganglia.

### The ancestors – MRJP8 and 9

The two genes *mrjp 8* and *9* showed a very different expression profile compared to the other seven *mrjps. Mrjp8* and *9* are not up-regulated in worker heads and expressed fair to high in the thorax and abdomen (Figure 1). Thus, both proteins are not tissue specific. Already Peiren et al. [50,51] showed that both proteins are not constrained to the food glands but also present in bee venom. In fact, MRJP8 has never been identified as component of RJ in *A. mellifera* and also MRJP9 was only identified in three out of numerous studies on the RJ proteome [20,24,25] alluding to a low amount of the protein in RJ. This is well in line with the previous notion that *mrjp1-7* derived from *mrjp8* and *9* during honeybee evolution [10]. Whereas *mrjp8* was consistently expressed in almost all analyzed samples, *mrjp9* was up-regulated in workers and virgin queens, compared to drones and mated queens (Figure 3, Table 1). Furthermore, *mrjp9* was the only *mrjp* with an expression higher than the reference gene in the thorax and the abdomen. As *mrjp9* shows a different expression profile, it clusters also in the dendrogram independent from all of the other *mrjps* (Figure 1). In addition, the MRJPs of species that possess just one MRJP (e.g., *Bombus terrestris*, *Camponotus floridanus, Harpegnathos saltator* and *Megachile rotundata*) show always the highest similarity (44 to 56%) to MRJP9 of the honeybee (Additional file 1: Table S4). This defines *mrjp9* as the most ancestral *mrjp* in *A. mellifera*, suggesting a completely different function for MRJP9 from the other MRJPs.

### Comparison across caste – nurses and foragers

There was a tremendous up-regulation of *mrjp1-7* in both the heads of nurses and foragers compared to the caged controls. The transcript abundances of *mrjp1-4* and *7* in nurses were higher than in foragers, although not always significantly higher (Figure 2). The lack of significance may have been due to the low sample size but also to the lack of age control in the foragers. Indeed, the variance for transcript abundance was significantly higher among the foragers than the nurse bees. We cannot exclude, that some foragers still had partially activated food glands which might have diluted the difference to the nurses. Although workers are typically considered as mature foragers if they carry pollen, there will be age variance among the analyzed foragers with potential residual food gland activity. Some workers may start foraging as early as day 10 (~10% of the time) [52]. Thus, foragers arriving with pollen at the hive may still be also performing nursing tasks within the hive. Since the caged bees were kept on a protein free diet and had no contact to brood, their hypopharyngeal glands were not activated [53,54] and did produce no or heavily reduced MRJPs 1–7. Clearly these bees showed that the hive context and the diet have a massive impact on *mrjp* transcripts and caged workers are important controls to visualize this effect.

In RJ, MRJP1, 2, 3 and 5 amount to 82% of total RJ proteins (31%, 16%, 26% and 9%, respectively) [9] and those four MRJPs are commonly identified with 1D-SDS-PAGE. This suggests that primarily MRJP1, 2, 3 and 5 have a function in RJ.

In this study, *mrjp3* was most strongly up-regulated in nurse bees (3,000-fold compared to caged workers and 130-fold compared to foragers). As the expression in all of the other analysed groups was only marginal, the protein seems to be particularly specific for young nurses and serves as important RJ ingredient. The nutritional function as food protein was already suggested for MRJP3 by Schmitzová et al. [9], especially because the protein contains a repetitive pentapeptide motive comprising several nitrogen rich amino acids [33]. MRJP3 was also associated with queen differentiation as specific post-translational modified isoforms of MRJP3 secreted into RJ lead to the development of queens [35].

Since also MRJP5 contains a repetitive motive with nitrogen rich amino acids, it has been considered as nutritional protein as well [9,32] and we found a 30-fold upregulation of *mrjp5* in nurse heads compared to caged workers_._ However, forager heads revealed a further 2-fold upregulation. Although this was not significant compared to the nurse bees, it nevertheless suggests that MRJP5 may have yet unknown functions in foragers beyond a plain food protein. Indeed, MRJP5 was detected in a significant higher amount in the brain proteome of foragers compared to nurses, whereas MRJP1-4 and 7 were higher in nurse brains [55] similar to our study.

*Mrjp6* was the only *mrjp* transcript which was significantly more abundant in foragers than in nurses (10-fold), suggesting again a function more specific to older worker bees. Both MRJP5 and 6 show the highest sequence identity (74%) among all MRJPs [10]. Since both genes also cluster together in the dendrogram based on *mrjp* expression levels (Figure 1a), it seems highly suggestive that both proteins are functionally important for foraging bees.

*Mrjp7* was 600-fold up-regulated in nurse heads compared to caged workers but is not a major component of RJ. Hojo et al. [44] found *mrjp7* to be one of the most abundant transcripts of the mushroom bodies in the honeybee brain. Furthermore, it was only marginally expressed in the hypopharyngeal glands [44]. These results point to a function of MRJP7 primarily in the brain.

Hojo et al. [44] also found *mrjp2* to be expressed in the mushroom bodies. In this study the expression level of *mrjp2* was very similar to the expression level of *mrjp3* in nurse bee heads. Since the amounts of these both proteins are very different in RJ (MRJP2 – 16%, MRJP3 – 26%, [9]) this supports the presence of an additional function of MRJP2 most likely in the brain.

MRJP1 is the most abundant protein of RJ [9] and shows with a relative expression of 226 in nurse bee heads by far the highest expression of all *mrjps* in any analyzed group. Since *mrjp1* expression is still elevated in foragers it is unlikely that the nutritional role and/or the possible role in queen differentiation are the only functions of MRJP1. The expression of *mrjp1* was already shown also in the brain [29,40-44] and more specifically in the Kenyon cells of the brain’s mushroom bodies, cells involved in the formation and storage of memory and learning [29].

### Comparison across sexuals – drones and queens

The expression patterns of *mrjps* are very similar among all sexuals and across all body sections (Figure 1). Most striking is that none of the *mrjps* showed any up-regulation in the heads. So whatever brain function MRJPs have in workers, the sexuals must do without it. Among queens, expression of *mrjp1* to *8* seems to be independent of queen age and mating. Virgin queens show a 5- to 30-fold up-regulation of *mrjp9* in all body parts. Nevertheless, the similarity of the expression levels among the queens is high and they form a tight cluster in the dendrogram, supported by high bootstrap values (Figure 1). Drones show barely any differences to mated queens but have a significant down-regulation of *mrjp9* compared to virgin queens. But as long as the functions of MRJP9 are not known, any functional interpretation remains pure speculation. Despite this small difference in *mrjp9* expression, drones show with a similarity distance of 3.76 a higher similarity to queens than to any analysed group of the worker caste (similarity distances above 4.4).

## Conclusions

To date, *mrjp* genes have been identified with different copy numbers in several species of the Hymenoptera, including the solitary parasitoid wasp *Nasonia vitripennis*, the leaf cutter ant *Atta cephalotes* and the buff-tailed bumblebee *Bombus terrestris* (for review see [10]). The high copy numbers in the genus *Apis* resulted from duplications of an ancient *mrjp* gene [7,10] and the results of the present study further confirm that this ancestor is *mrjp9*.

Although the influence of caste is much higher on *mrjp* expression than the influence of sex, we can show that *mrjps* are widely expressed in all castes and both sexes and not limited to the food glands of nurse bees. Queens and drones show almost the same expression patterns for all *mrjps*. Thus, both sexes cluster together in a discrete branch in the group-tree based on *mrjp* expression (Figure 1). Furthermore, the sexuals are more similar to the caged controls (similarity distance 4.44) than to the other two worker castes (similarity distance 6.66), due to the head expression of *mrjp1-7* in foragers and hive nurses. So clearly MRJP1-7 are typical worker caste proteins connected to labour in the colony. But even in the worker caste, functions of the proteins differ and some MRJPs, like MRJP1 and 2, are obviously polyfunctional.

In spite of this dominating and clear cut differentiation in the female caste, all of our results concerning *mrjp* expression in workers suggest that MRJPs not only have a nutritional function in royal jelly, but also a more general physiological one for all organisms in the colony. In particular, the evolutionary old *mrjp9* and *mrjp8* cannot serve as food proteins since neither queens nor drones feed anybody in the colony. Furthermore, the consistent expression across all body sections indicates a more general physiological role. For the sole MRJP in *B. terrestris* a non-nutritive function was already suggested before [12]. The picture of *mrjp*s in honeybees remains complex and albeit feeding larvae represents without doubt a function for some MRJPs others have clearly profound roles in the brain and fulfill tasks that still have to be elucidated.

## Material and methods

### Honeybee samples

Honeybees (*Apis mellifera*) were sampled in June and July 2013 from the University apiary. Drones (D) and pollen foragers (F) were caught directly at the flight entrance of one colony and frozen in liquid nitrogen.

To rear nurse bees, a brood frame was removed from the hive and incubated at 34°C and ~60% relative humidity until the bees hatched. Freshly emerged workers were paint marked and returned to the hive (nurse bees (N)). To test for the effect of age, we also reared freshly emerged workers in hoarding cages in the incubator fed with a non-protein diet (79% powdered sugar (w/w), 20% honey (w/w)) *ad libitum* to obtain equally aged workers but without developed food glands (caged workers (C)) [53]. After four days, caged workers and hive nurses were freeze killed in liquid nitrogen.

Queen bees were raised from queen cells with the help of nurse bees in incubators at 34°C and ~60% humidity. Virgin queens (Q_V_) were freeze killed a few days after hatching. For each group (drones, caged workers, nurses, foragers and virgin queens) nine bees were collected and stored at -80°C until further processing. Queens destined to be mated were introduced into mating hives with ~2000 worker bees and allowed to perform mating flights. Three of the mated queens were freeze killed directly after initiation of oviposition and the production of first eggs (Q_E_) whereas three other queens (Q_P_) were freeze killed until the first pupae had developed (~ 15 d after first oviposition - confirmed by white worker pupae with red eyes).

### Gene expression

Total RNA was extracted from the head, thorax and abdomen of nine individuals of each group (three for the two mated queen groups) using the RNeasy Mini Kit (Qiagen, Hilden, Germany) according to the manufacture’s protocol. Quality and quantity of the total RNA were photometrically determined with a NanoDrop 1000 (Thermo Fisher Scientific, Wilmington, DE, USA). 500 ng total RNA were reverse transcribed using 0.4 μg Oligo (dT)_15_ Primer (Promega, Mannheim, Germany), 0.8 μl dNTPs (10 mM) and 80 U M-MLV reverse transcriptase (Promega, Mannheim, Germany). cDNA was purified with the QIAquick PCR Purification Kit (Qiagen, Hilden, Germany) as described in the manufacture’s protocol and the concentration set to 15 ng/μl. cDNA of mated queens was used directly for quantitative real-time PCR (qPCR) analyses whereas the cDNA of all other groups was pooled to minimize individual variation from three individuals, i.e. body sections, to one pool. Finally three pools per body section and group, except for mated queens, were used.

For qPCR reactions, 1 μl cDNA (15 ng/μl) was mixed with 5 μl SensiMixPlus SYBR & Fluorescein Kit (Bioline, Luckenwalde, Germany), 0.3 μM of each gene specific primer and 3.4 μl DEPC-water. Gene specific primers were designed to span at least one intron using sequences of *Apis mellifera mrjp1-9* that were available on GenBank in May 2013 and the programme Primer-BLAST of the National Center for Biotechnology Information (NCBI) (Additional file 1: Table S1). *Ribosomal protein S5a* (*RpS5a*) and *actin related protein 1 (arp1*, also known as *Actin)* were initially chosen in order to standardize expression levels between individuals and groups [56,57].

The same qPCR protocol was used for all primer pairs. An initial denaturation step of 10 min at 95°C was followed by 40 amplification cycles (95°C, 15 sec; 57°C, 30 sec; 72°C, 30 sec), and a subsequent melting curve analysis between 55°C and 98°C, reading the fluorescence at 1°C increments. Two technical replicates were run for each sample using Chromo4™ (Bio-Rad, Munich, Germany) and repeated if necessary, to reach at maximum an in between replicate threshold cycle (C_t_) difference of 0.5.

### Statistics

LinRegPCR version 12.10 [58] was used to determine the C_t_ values after baseline subtraction. PCR efficiency for each target gene was estimated by serial dilution qPCR (Additional file 1: Table S1) and relative target gene expression was determined as described [59] using *RpS5a *[57] as honeybee reference gene (Table 1). Heat maps combined with dendrograms were used to show gene and caste specific expression differences. We performed a hierarchical clustering analysis using the software MultiExperiment Viewer (MeV) version 4.9. Within this analysis, a dendrogram tree was built for each group (samples, genes) based on the raw gene expression data. Furthermore, we used the option ‘optimized gene and caste leaf order’ pending on the data set. To build the heat map and dendrogram the Euclidean distance between samples and genes and single linkage clustering as clustering method were used to build the final figure [60]. The Clustering Calculator software (http://www2.biology.ualberta.ca/jbrzusto/cluster.php) was used with the same settings as mentioned above to verify the topology of the inferred dendrogram (bootstrap resampling, 1000 replications), and to calculate similarity distances.

All statistical analyses were performed with STATISTICA 8.0 (StatSoft, Tulsa, OK, USA). Data were tested for deviations form a normal distribution by Kolmogorov-Smirnov tests and log- or Box-Cox-transformed in the case of significant deviations from normality and homoscedasticity. *Mrjp* gene specific comparisons between and within groups (e.g. caste and/or body section) were done using one-way analysis of variance (ANOVA) with Bonferroni post-hoc test. Statistical details for all groups and genes are given in the Additional file 2.

## Abbreviations

C: Caged workers; D: Drones; F: Foragers; MRJP: Major royal jelly protein; N: Nurses; QE: Queens with eggs; QP: Queens with pupae; QV: Virgin queens; RJ: Royal jelly.

## Competing interests

The authors declare that they have no competing interests.

## Authors’ contributions

AB, RFAM and SE conceived the study and designed the experimental set-up. AB executed the experiments. AB and SE analysed the data. AB wrote the first draft of the manuscript. All authors read and approved the final manuscript.

## Supplementary Material

Additional file 1: Figure S1 Analysis of qPCR product specificity; *mrjp1* – 89 bp, *mrjp2* – 90 bp, *mrjp3* – 102 bp, *mrjp4* – 91 bp, *mrjp5* – 80 bp, *mrjp6* – 146 bp, *mrjp7* – 95 bp, *mrjp8* – 130 bp, *mrjp9* – 103 bp, *RpSa5* – 115 bp, *arp1* – 120 bp. **Figure S2.** Two-way hierarchical clustering analysis heat map and dendrogram of *mrjp* gene expression data over all honeybee castes and groups. Branch lengths in dendrograms produced from cluster analysis correspond to the relative degree of similarity between branches. Differential gene expression is represented for all genes as a color gradient across all samples from deep blue (lowest) to light yellow (highest). The expression values were log transformed and visualized using MultiExperiment Viewer (MeV) version 4.9. **Table S1.** Primer characteristics for qPCR. Primer sequences for *RpS5a* and *arp1* were adopted from [56] and [57]. **Table S2.** Expression profile of a specific *major royal jelly protein* over all analyzed groups within honeybee body sections. **Table S3.** Expression profiles of all *major royal jelly proteins* in a specific group within honeybee body sections. **Table S4.** Amino acid sequence identities of all *Apis mellifera* MRJPs and the single MRJPs of *Bombus terrestris* (ADW82102.1), *Megachile rotundata* (XP_003708472.1), *Camponotus floridanus* (EFN61808.1) and *Harpegnathos saltator* (EFN81209.1). Sequence identities were determined using ClustalW2 (http://www.ebi.ac.uk/Tools/msa/clustalw2/). Click here for file

Additional file 2 Statistical details for Tables S2 and S3. Click here for file
