# Supplementary material for: More than royal food - Major royal jelly protein genes in sexuals and workers of the honeybee Apis mellifera
Source: Front Zool. 2013 Nov 27;10:72. doi: 10.1186/1742-9994-10-72 (PMC4176732; doi:10.1186/1742-9994-10-72)
Supplement: Additional file 1: Figure S1 — Analysis of qPCR product specificity; mrjp1 – 89 bp, mrjp2 – 90 bp, mrjp3 – 102 bp, mrjp4 – 91 bp, mrjp5 – 80 bp, mrjp6 – 146 bp, mrjp7 – 95 bp, mrjp8 – 130 bp, mrjp9 – 103 bp, RpSa5 – 115 bp, arp1 – 120 bp. Figure S2. Two-way hierarchical clustering analysis heat map and dendrogram of mrjp gene expression data over all honeybee castes and groups. Branch lengths in dendrograms produced from cluster analysis correspond to the relative degree of similarity between branches. Differential gene expression is represented for all genes as a color gradient across all samples from deep blue (lowest) to light yellow (highest). The expression values were log transformed and visualized using MultiExperiment Viewer (MeV) version 4.9. Table S1. Primer characteristics for qPCR. Primer sequences for RpS5a and arp1 were adopted from [56] and [57]. Table S2. Expression profile of a specific major royal jelly protein over all analyzed groups within honeybee body sections. Table S3. Expression profiles of all major royal jelly proteins in a specific group within honeybee body sections. Table S4. Amino acid sequence identities of all Apis mellifera MRJPs and the single MRJPs of Bombus terrestris (ADW82102.1), Megachile rotundata (XP_003708472.1), Camponotus floridanus (EFN61808.1) and Harpegnathos saltator (EFN81209.1). Sequence identities were determined using ClustalW2 (http://www.ebi.ac.uk/Tools/msa/clustalw2/). [file 1742-9994-10-72-S1.pdf]

Additional File 1

Buttstedt A, Moritz RFA & Erler S (2013): More than royal food - *Major royal jelly protein* genes in sexuals and workers of the honeybee *Apis mellifera* – Frontiers in Zoology

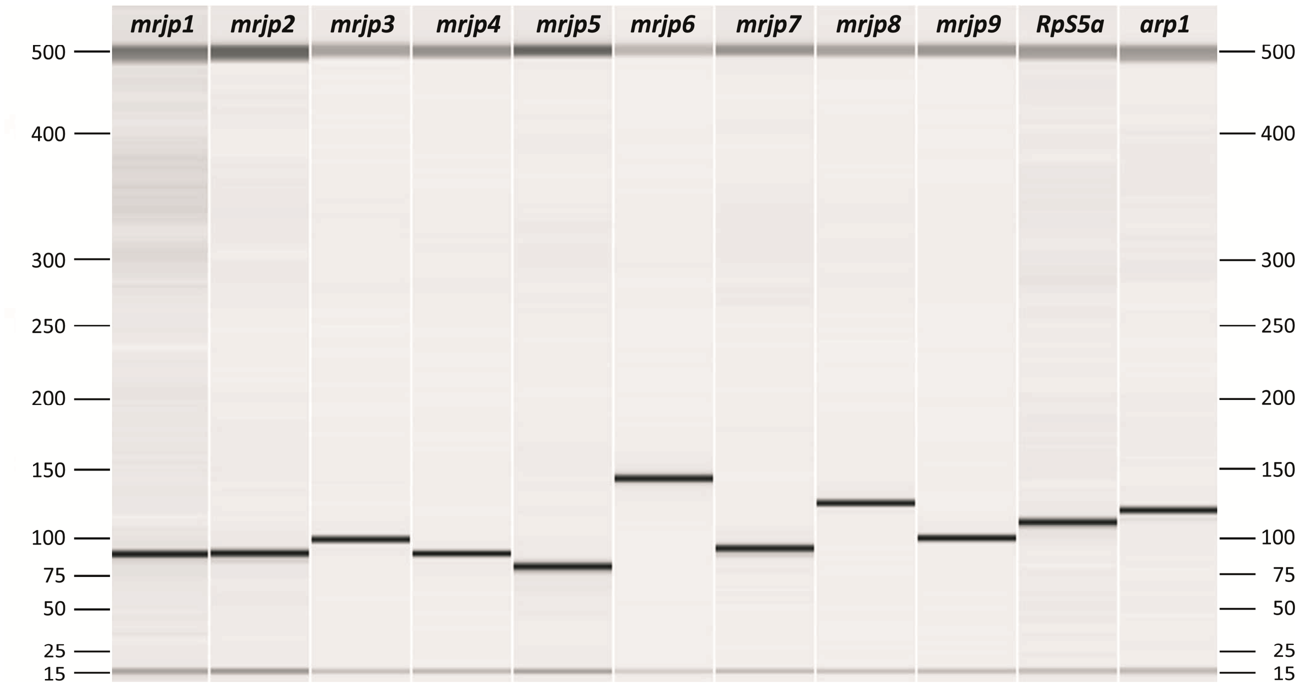

Figure S1: Analysis of qPCR product specificity; *mrjp1* – 89 bp, *mrjp2* – 90 bp, *mrjp3* – 102 bp, *mrjp4* – 91 bp, *mrjp5* – 80 bp, *mrjp6* – 146 bp, *mrjp7* – 95 bp, *mrjp8* – 130 bp, *mrjp9* – 103 bp, *RpSa5* – 115 bp, *arp1* – 120 bp.

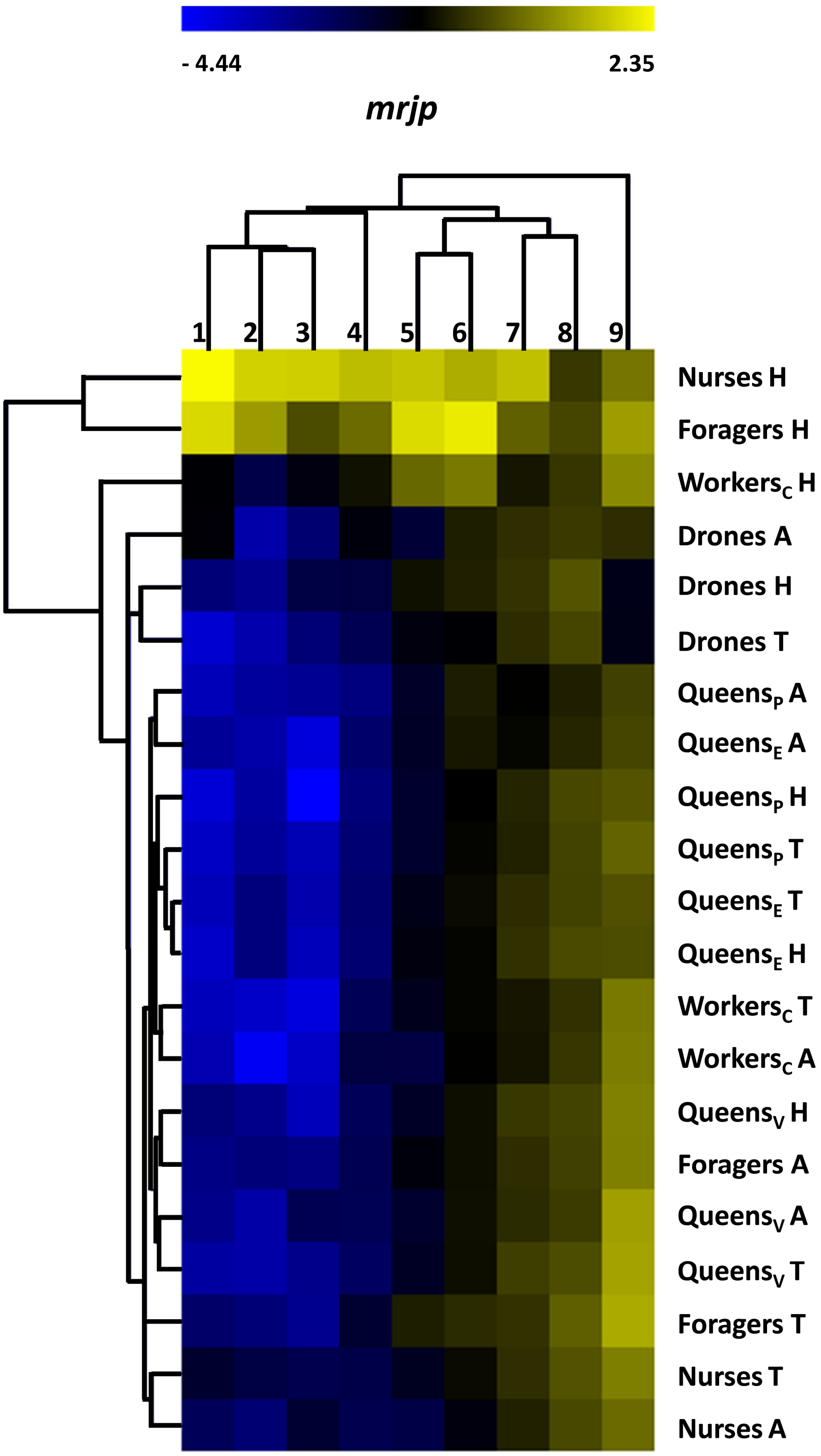

Figure S2: Two-way hierarchical clustering analysis heat map and dendrogram of *mrjp* gene expression data over all honeybee castes and groups. Branch lengths in dendrograms produced from cluster analysis correspond to the relative degree of similarity between branches. Differential gene expression is represented for all genes as a color gradient across all samples from deep blue (lowest) to light yellow (highest). The expression values were log transformed and visualized using MultiExperiment Viewer (MeV) version 4.9.

Table S1: Primer characteristics for qPCR. Primer sequences for *RpS5a* and *arp1* were adopted from Evans (2006) and Gregorc *et al*., (2012).

Name		Primer Sequence (5'-3')	Size (bp)	Product T_m (°C)	PCR efficiency	GenBank Gene ID
mrjp1	F	TGACATACATTACGAAGGAGTCCA	89	76.5	2.04	406090
	R	ATCCGAAGAAGAGAACGCCA				
mrjp2	F	CGTCCAATACCAAGGATCCGAA	90	78.0	2.01	406091
	R	ACAAGTCCGACGAAGAGGAC				
mrjp3	F	TGGACAGATGGCGTGATAAGAC	102	77.0	1.96	406120
	R	GAGGTCCACCTTTGCCCTTT				
mrjp4	F	AGACAAAATATCGATGTCGTAGCTC	91	77.0	2.04	406133
	R	TGCCAGATTGTGGAACGTTTT				
mrjp5	F	AGAAAATATGGATATGGTCGCTCAG	80	76.7	1.91	406116
	R	ATTGTGGGAGATGCATCATTTTCA				
mrjp6	F	ACAAAATATGGATATGGTCGCTCAG	146	75.0	1.90	406149
	R	TCTGCATTCTGTTACTTAAAGCCAA				
mrjp7	F	AGAAAATACCGATATGGTCGCC	95	76.0	2.01	409555
	R	CAATAATGACGATATGTGGGAGCA				
mrjp8	F	TGGACTCAAGCATCGGCTAA	130	79.0	1.99	406067
	R	TGGCAACCACTTCGATATTTCTT				
mrjp9	F	TCGGAAACTGGTGCTCTCTT	103	80.0	1.85	409873
	R	TGGCGACTATTTCAATATTCCTTCT				
RpS5a	F	AATTATTTGGTCGCTGGAATTG	115	75.0	2.07	409728
	R	TAACGTCCAGCAGAATGTGGTA				
arp1	F	TTGTATGCCAACACTGTCCTTT	120	79.0	1.91	406122
	R	TGGCGCGATGATCTTAATTT				

Table S2: Expression profile of a specific *major royal jelly protein* over all analyzed groups within honeybee body sections.

Relative *mrjp* gene expression within body sections

	Head						
mrjp1	N^{a}	F^{a}	C^{b}	D^{c}	Q_{V}^{c}	Q_{E}^{c}	Q_{P}^{c}
mrjp2	N^{a}	F^{a}	C^{b}	$Q_{E}^{b,c}$	Q_{V}^{c}	$D^{b,c}$	Q_{P}^{c}
mrjp3	N^{a}	F^{b}	C^{c}	$D^{c,d}$	$Q_{V}^{d,e}$	$Q_{E}^{d,e}$	Q_{P}^{e}
mrjp4	N^{a}	F^{b}	C^{c}	D^{d}	Q_{V}^{d}	Q_{E}^{d}	Q_{P}^{d}
mrjp5	F^{a}	N^{a}	C^{b}	D^{c}	$Q_{E}^{c,d}$	Q_{V}^{d}	Q_{P}^{d}
mrjp6	F^{a}	N^{b}	C^{c}	D^{d}	$Q_{V}^{d,e}$	$Q_{E}^{d,e}$	Q_{P}^{e}
mrjp7	N^{a}	$F^{a,b}$	$Q_{V}^{b,c}$	$D^{b,c}$	$Q_{E}^{b,c}$	$Q_{P}^{c,d}$	C^{d}
mrjp8	D^{a}	$Q_{E}^{a,b}$	Q_{P}^{a-c}	$F^{b,c}$	$Q_{V}^{b,c}$	$N^{b,c}$	C^{c}
mrjp9	F^{a}	C^{b}	$Q_{V}^{b,c}$	N^{b-d}	$Q_{P}^{c,d}$	Q_{E}^{d}	D^{d}

	Thorax						
mrjp1	N^{a}	F^{b}	$Q_{V}^{b,c}$	Q_{E}^{c}	C^{c}	Q_{P}^{c}	D^{c}
mrjp2	N^{a}	$F^{a,b}$	$Q_{E}^{a,b}$	$Q_{P}^{b,c}$	$Q_{V}^{b,c}$	$D^{b,c}$	C^{c}
mrjp3	N^{a}	$D^{a,b}$	$Q_{V}^{a,b}$	$F^{a,b}$	$Q_{E}^{a,b}$	$Q_{P}^{a,b}$	C^{b}
mrjp4	F^{a}	$N^{a,b}$	D^{b}	C^{b}	Q_{V}^{b}	Q_{E}^{b}	Q_{P}^{b}
mrjp5	F^{a}	$D^{a,b}$	Q_{E}^{b}	C^{b}	N^{b}	Q_{V}^{b}	Q_{P}^{b}
mrjp6	F^{a}	$Q_{V}^{a,b}$	$Q_{E}^{a,b}$	N^{b}	Q_{P}^{b}	C^{b}	D^{b}
mrjp7	Q_{V}^{a}	$F^{a,b}$	$N^{a,b}$	$Q_{E}^{a,b}$	$D^{a,b}$	Q_{P}^{b}	C^{b}
mrjp8	F^{a}	$N^{a,b}$	$Q_{V}^{a,b}$	D^{b}	Q_{P}^{b}	Q_{E}^{b}	C^{b}
mrjp9	F^{a}	Q_{V}^{a}	N^{b}	$C^{b,c}$	$Q_{P}^{c,d}$	Q_{E}^{d}	D^{e}

	Abdomen						
mrjp1	D^{a}	$N^{a,b}$	$F^{a,b}$	$Q_{V}^{a,b}$	Q_{E}^{b}	C^{b}	Q_{P}^{b}
mrjp2	N^{a}	F^{a}	Q_{P}^{a}	Q_{V}^{a}	Q_{E}^{a}	D^{a}	C^{a}
mrjp3	N^{a}	Q_{V}^{b}	D^{b}	F^{b}	Q_{P}^{b}	C^{b}	Q_{E}^{b}
mrjp4	D^{a}	$C^{a,b}$	$N^{b,c}$	$F^{b,c}$	$Q_{V}^{b,c}$	$Q_{E}^{b,c}$	Q_{P}^{c}
mrjp5	F^{a}	Q_{E}^{a}	Q_{P}^{a}	Q_{V}^{a}	D^{a}	C^{a}	N^{a}
mrjp6	D^{a}	Q_{P}^{a}	Q_{E}^{a}	Q_{V}^{a}	F^{a}	C^{a}	N^{a}
mrjp7	D^{a}	F^{a}	$Q_{V}^{a,b}$	$N^{a,b}$	$C^{a,b}$	Q_{E}^{b}	Q_{P}^{b}
mrjp8	N^{a}	$F^{a,b}$	$Q_{V}^{a,b}$	$D^{a,b}$	$C^{a,b}$	Q_{E}^{b}	Q_{P}^{b}
mrjp9	Q_{V}^{a}	F^{b}	$C^{b,c}$	$N^{c,d}$	Q_{E}^{d}	Q_{P}^{d}	D^{d}

C, caged workers; N, hive nurses; F, foragers; D, drones; Q_V, virgin queens; Q_E, queens with eggs; Q_P, queens with pupae. Data were analyzed with one-way ANOVA followed by Bonferroni post-hoc test. Data that did not match normality untransformed, were log- or Box-Cox-transformed. $^{a-e}$Gene expression of groups in the same row with different superscripts is significantly different ($P < 0.05$). Genes are listed according to their mean expression values from large to small. Relative gene expression levels are given in Table 1 of the main manuscript and statistical details for all groups and genes in additional file 2.

Table S3: Expression profiles of all *major royal jelly proteins* in a specific group within honeybee body sections.

	Relative *mrjp* gene expression within body sections																										
	Head									Thorax									Abdomen								
**C**	9^{a}	6^{a}	5^{a}	8^{b}	$7^{b,c}$	4^{b-d}	$1^{c,d}$	$3^{d,e}$	2^{e}	9^{a}	8^{b}	$7^{b,c}$	$6^{b,c}$	$5^{c,d}$	4^{d}	1^{e}	2^{e}	3^{e}	9^{a}	8^{b}	7^{c}	6^{c}	4^{d}	5^{d}	1^{e}	3^{e}	2^{e}
**N**	1^{a}	2^{b}	3^{b}	$5^{b,c}$	$7^{b,c}$	$4^{b,c}$	6^{c}	9^{d}	8^{e}	9^{a}	$8^{a,b}$	$7^{b,c}$	$6^{c,d}$	$5^{d,e}$	$1^{d,e}$	2^{e}	4^{e}	3^{e}	9^{a}	8^{b}	7^{c}	6^{d}	$3^{d,e}$	$5^{e,f}$	$4^{e,f}$	$1^{e,f}$	2^{f}
**F**	6^{a}	$5^{a,b}$	$1^{a,b}$	$9^{b,c}$	$2^{b,c}$	$4^{c,d}$	$7^{c,d}$	3^{d}	8^{d}	9^{a}	8^{b}	7^{c}	6^{c}	5^{c}	4^{d}	1^{e}	2^{e}	3^{e}	9^{a}	8^{b}	7^{b}	6^{c}	5^{d}	4^{e}	2^{f}	3^{f}	1^{f}
**D**	8^{a}	$7^{a,b}$	6^{a-c}	5^{b-d}	9^{c-e}	4^{d-f}	$3^{e,f}$	1^{f}	2^{f}	8^{a}	$7^{a,b}$	$6^{b,c}$	$5^{b,c}$	$9^{b,c}$	$4^{c,d}$	$3^{d,e}$	$2^{d,e}$	1^{e}	8^{a}	$7^{a,b}$	$9^{a,b}$	$6^{b,c}$	1^{c}	4^{c}	5^{c}	3^{c}	2^{c}
**Q_V**	9^{a}	8^{b}	7^{b}	$6^{b,c}$	$5^{c,d}$	$4^{d,e}$	$1^{e,f}$	$2^{e,f}$	3^{f}	9^{a}	8^{b}	7^{b}	6^{c}	5^{d}	4^{e}	3^{f}	1^{f}	2^{f}	9^{a}	8^{b}	7^{b}	6^{c}	5^{d}	3^{e}	4^{e}	1^{f}	2^{g}
**Q_E**	9^{a}	8^{a}	7^{a}	6^{b}	5^{b}	4^{c}	2^{c}	3^{d}	1^{d}	9^{a}	$8^{a,b}$	7^{a-c}	$6^{b,c}$	$5^{c,d}$	$4^{d,e}$	$2^{d,e}$	3^{e}	1^{e}	9^{a}	$8^{a,b}$	6^{a-c}	7^{a-c}	5^{b-d}	4^{b-d}	$1^{c,d}$	$2^{c,d}$	3^{d}
**Q_P**	9^{a}	8^{a}	$7^{a,b}$	$6^{b,c}$	5^{c}	4^{d}	$2^{d,e}$	$1^{e,f}$	3^{f}	9^{a}	8^{a}	7^{b}	6^{b}	5^{c}	4^{d}	$2^{d,e}$	3^{e}	1^{e}	9^{a}	$8^{a,b}$	6^{b}	$7^{b,c}$	5^{c}	4^{d}	3^{d}	2^{d}	1^{d}

C, caged workers; N, hive nurses; F, foragers; D, drones; Q_V, virgin queens; Q_E, queens with eggs; Q_P, queens with pupae. Data were analyzed with one-way ANOVA followed by Bonferroni post-hoc test. Data that did not match normality untransformed, were log- or Box-Cox-transformed. $^{a-g}$ Gene expression of *mrjps* in the same row with different superscripts is significantly different (P < 0.05). Genes are listed according to their mean expression values from large to small. Relative gene expression levels are given in Table 1 of the main manuscript and statistical details for all groups and genes in additional file 2.

Table S4: Amino acid sequence identities of all *Apis mellifera* MRJPs and the single MRJPs of *Bombus terrestris* (GenBank: ADW82102.1), *Megachile rotundata* (GenBank: XP_003708472.1), *Camponotus floridanus* (GenBank: EFN61808.1) and *Harpegnathos saltator* (GenBank: EFN81209.1). Sequence identities were determined using ClustalW2 (http://www.ebi.ac.uk/Tools/msa/clustalw2/).

	B. terrestris	*M. rotundata*	*C. floridanus*	*H. saltator*
Am_MRJP1	48.9%	46.1%	39.3%	36.3%
Am_MRJP2	48.9%	43.9%	39.3%	36.3%
Am_MRJP3	46.7%	44.6%	37.9%	35.8%
Am_MRJP4	42.6%	38.7%	33.7%	31.5%
Am_MRJP5	47.5%	43.6%	39.3%	36.3%
Am_MRJP6	46.2%	45.4%	40.6%	37.8%
Am_MRJP7	48.4%	46.1%	41.6%	38.0%
Am_MRJP8	52.3%	46.4%	44.0%	41.3%
Am_MRJP9	55.5%	49.6%	46.4%	44.0%
